# Supplementary material for: Symmetry-controlled edge states in the type-II phase of Dirac photonic lattices
Source: Nat Commun. 2020 Apr 29;11:2074. doi: 10.1038/s41467-020-15952-z (PMC7190735; doi:10.1038/s41467-020-15952-z)
Supplement: Supplementary file 1 — Supplementary Information [file 41467_2020_15952_MOESM1_ESM.pdf]

Supplementary Information for:

Symmetry-controlled edge states in the type-II phase of Dirac photonic lattices

Pyrialakos et al.

Supplementary Note 1: Effective 2×2 Hamiltonian of the anisotropic lattice

To demonstrate how the anisotropic square lattice is related to the Dirac model of equation (1) and how the $t_{1-4,s}$ terms define its properties, consider the following Hamiltonian acting on $[\upsilon_A, \upsilon_B]$ of Fig. 1

$$H(\mathbf{k}) = \begin{bmatrix} h_{11} & h_{12} \\ h_{21} & h_{22} \end{bmatrix}, \tag{1}$$

$$h_{11} = h_{22} = t_s e^{ik_y} + t_s e^{-ik_y} = 2t_s \cos k_y, \tag{2}$$

$$\begin{aligned} h_{12} = h_{21}^* &= t_1 e^{\frac{i(k_x+k_y)}{2}} + t_2 e^{\frac{i(k_x-k_y)}{2}} + t_3 e^{-\frac{i(k_x+k_y)}{2}} + t_4 e^{-\frac{i(k_x-k_y)}{2}} \\ &= (t_1 + t_3)\cos\left(\frac{k_x+k_y}{2}\right) + (t_4 + t_2)\cos\left(\frac{k_x-k_y}{2}\right) + i(t_1 - t_3)\sin\left(\frac{k_x+k_y}{2}\right) + i(t_4 - t_2)\sin\left(\frac{k_x-k_y}{2}\right), \end{aligned} \tag{3}$$

where the length of the square unit cell has been normalized to unity. The presence of Dirac cones in momentum space can be determined by imposing the condition $h_{12}(\mathbf{k}_\mathrm{D}) = 0$, searching for individual $\mathbf{k}_\mathrm{D} = (k_x^\mathrm{D}, k_y^\mathrm{D})$ points where the two eigen-solutions of the 2×2 system meet (excluding trivial lines in $\mathbf{k}$ where they would instead intersect). For this lattice, these points are given by

$$k_x^\mathrm{D} = 2\arctan\left[\sqrt{-\frac{(t_3+t_4)^2-(t_1+t_2)^2}{(t_3-t_4)^2-(t_1-t_2)^2}}\right], \tag{4}$$

$$k_y^\mathrm{D} = 2\arctan\left[-\sqrt{\frac{(t_3+t_2)^2-(t_1+t_4)^2}{(t_3-t_2)^2-(t_1-t_4)^2}}\right]. \tag{5}$$

Valid solutions can be obtained when the terms under the square roots are positive. Then, the velocities of the u^T vector and the elements of the u^D matrix will be given by

$$u^\mathrm{T} = -2t_s \sin k_y^\mathrm{D}, \tag{6}$$

$$u_{x,x}^\mathrm{D} = 2(t_3 + t_1)\sin\left(\frac{k_x^\mathrm{D}+k_y^\mathrm{D}}{2}\right) + 2(t_2 + t_4)\sin\left(\frac{k_x^\mathrm{D}-k_y^\mathrm{D}}{2}\right), \tag{7}$$

$$u_{y,x}^\mathrm{D} = 2(t_3 + t_1)\sin\left(\frac{k_x^\mathrm{D}+k_y^\mathrm{D}}{2}\right) - 2(t_2 + t_4)\sin\left(\frac{k_x^\mathrm{D}-k_y^\mathrm{D}}{2}\right), \tag{8}$$

$$u_{x,y}^\mathrm{D} = 2(t_3 - t_1)\cos\left(\frac{k_x^\mathrm{D}+k_y^\mathrm{D}}{2}\right) + 2(t_2 - t_4)\cos\left(\frac{k_x^\mathrm{D}-k_y^\mathrm{D}}{2}\right), \tag{9}$$

$$u_{y,y}^\mathrm{D} = 2(t_3 - t_1)\cos\left(\frac{k_x^\mathrm{D}+k_y^\mathrm{D}}{2}\right) - 2(t_2 - t_4)\cos\left(\frac{k_x^\mathrm{D}-k_y^\mathrm{D}}{2}\right), \tag{10}$$

applying the proper derivatives to Supplementary Equation 3. In essence, around the $\mathbf{k}_\mathrm{D}$ points we can express Supplementary Equation 1 under the base of Pauli matrices and determine a one-to-one correspondence between the parameters of the relativistic model $(u^\mathrm{D}, u^\mathrm{T})$ of equation (1) and the hoping terms of the square lattice.

The effective 2×2 Dirac representation of Supplementary Equation 1 can be also extracted for the multi-element square lattice. To demonstrate this process, we separate each individual chain and reduce the rank of its matrix form in the tight-binding approximation. This implies independence between the chains, i.e. all elements in a particular chain should only communicate with elements of the same chain or the central nodes of the lattice. In essence, four sequential elements (Supplementary Figure 1a) can be represented by the next system

$$k_z|\upsilon\rangle = \begin{bmatrix} V_2 & t_{12} & 0 & 0 \\ t_{12} & V_1 & t_{11} & 0 \\ 0 & t_{11} & V_1 & t_{12} \\ 0 & 0 & t_{12} & V_2 \end{bmatrix} \begin{bmatrix} u_{-2} \\ u_{-1} \\ u_1 \\ u_2 \end{bmatrix}, \tag{11}$$

where we have imposed equivalence between the symmetric pairs in order to fulfill parity invariance. We, now, reduce the 4×4 system by substituting the two central rows of the matrix to the first and fourth row, leading to

$$\beta|\upsilon\rangle = \begin{bmatrix} V_{22} & t_{22} \\ t_{22} & V_{22} \end{bmatrix} \begin{bmatrix} \upsilon_{-2} \\ \upsilon_2 \end{bmatrix}, \tag{12}$$

$$V_{22} = (k_z - V_2) - \frac{t_{12}(k_z - V_1)}{(k_z - V_1)^2 - t_{11}^2}, \tag{13}$$

$$t_{22} = \frac{t_{12}^2 t_{11}}{(\beta - V_1)^2 - t_{11}^2}. \tag{14}$$

To transform the new system into a valid eigenproblem, we should remove eigenvalue k_z from the reduced Hamiltonian. Hence, let us assume the existence of a Dirac degeneracy at the eigenvalue $k_z = k_z^{\mathrm{D}}$ and apply the proper transformation,

$$e^{\beta' t} = e^{(k_z - k_z^{\mathrm{D}})t}, \tag{15}$$

to the solution of Supplementary Equation 11, before reaching Supplementary Equations 12-14. Assuming a small region around $(k_x^{\mathrm{D}}, k_y^{\mathrm{D}})$, where $k_z = k_z^{\mathrm{D}}$, the effective 2×2 Hamiltonian will, now, be generated by the subsequent elements

$$V_{22} = V_2 - \frac{t_{12} V_1}{V_1^2 - t_{11}^2},. \tag{16}$$

$$t_{22} = \frac{t_{12}^2 t_{11}}{V_1^2 - t_{11}^2}. \tag{17}$$

In general, the transformation of Supplementary Equation 15 will not nullify the self-potentials V_1 and V_2, unless all nodes are equivalent. Regardless, we can observe that the effective t_{22} hopping term will increase or decrease in magnitude by a corresponding change of the boding strength between the four elements (i.e. a concurrent variation of the t_{11}, t_{12}, and V_1 variables). By extending this procedure to six adjacent sites and substituting t_{11} with t_{22} as well as t_{12} with t_{23} in Supplementary Equations 16 and 17, we can derive a similar expression for t_{33},

$$t_{33} = \frac{t_{23}^2 t_{22}}{V_2^2 - t_{22}^2}. \tag{18}$$

In the square lattice of Supplementary Figure 1a, waveguide 3 is associated with either site A or site B, while t_{33} may correspond to any of the four diagonal hoping terms ($t_{33} = t_{\mathrm{AB}} = t_{1-4}$). This indicates an explicit relationship between the magnitudes of t_{1-4} and the combined magnitudes of the hoping variables that define each chain (i.e. t_{11}, t_{12} and $t_{23} = t_{2\mathrm{A}} = t_{2\mathrm{B}}$).

In a similar fashion, by adding six elements between sites A-A and sites B-B (for a total of 8 elements in each chain), along the horizontal or vertical direction, we construct the next-nearest-neighbor hoping terms t_s (e.g. in Supplementary Figure 1f). Ultimately, all 5 hoping terms will be exclusively related to their own unique set of variables. Through this property, the chained lattice overcomes the fundamental limitations of the two-atom model (namely, a square lattice with direct couplings between sites A and B), in which the magnitudes of t_{1-4} are entangled for every gap-preserving perturbation, whereas t_s is realistically always negligible[1]. If a proper realization of this tight-binding model is achieved, one can then attain full control over equation (1) and the extended Dirac model.

The real set of solutions produced by Supplementary Equations 4 and 5 spans the entire first Brillouin zone and are not restricted to the high symmetry lines (in distinction to the honeycomb lattice). However, the **k** space naturally splits into two equal sections, as shown in Supplementary Figure 1b, where for the inner section, valid solutions require at least one negative hoping term. In the chained lattice all effective terms maintain a positive sign regardless of the magnitude of detuning in the middle elements. Therefore, the Dirac points can only be positioned in the outer section of Supplementary Figure 1b (if detuning is introduced in all four chains).

The chain methodology introduced herein, hints a possibility to attain coexisting negative and positive hoping terms. Actually, in a lattice where all elements are identical, the self-potential terms of the matrix in Supplementary Equation 11 vanish. This leads to a set of more simplified equation for the effective t_{22} term (Supplementary Equations 16 and 17), now, given by

$$V_{22} = 0, \tag{19}$$

$$t_{22} = -t_{11} = -t_{12}, \tag{20}$$

where the sign of t_{22} is flipped. By repeating the process (with the addition of two more elements in the chain and the definition of the t_{33} hoping term), the sign flips again ($t_{33} = -t_{22}$) as long as the same properties are maintained (element self-potential

[1]Essentially, to maintain a gapless state and the presence of Dirac cones in momentum space, the self-potential terms of A and B must remain balanced ($V_{\mathrm{A}} = V_{\mathrm{B}}$). As a result, in the two-atom model, all perturbations to t_{1-4} must result from either direct movement of the A and B sites or from variations of the unit cell itself (deviating from the square lattice topology).

and distance between elements). In Supplementary Figure 1c, we theorize a lattice with 6 elements in the fourth connective chain, two more over the other three, which results in the band diagram of Supplementary Figure 1d, for the middle, symmetric pair of bands. This outcome resembles the band diagram of the anisotropic tight-binding model ($t_1, t_2, t_3, t_4 = 1,1,1,-1$); Supplementary Figure 1e), which, according to Supplementary Equations 10, produces a pair of isotropic Dirac cones at ($\pm\pi/2, \pm\pi/2$). From a topological viewpoint, this model does not offer any benefit over the three-chain model, while possibly presenting higher fabrication complexity and sensitivity to perturbation. Nonetheless, breaking the barrier towards the center region of Supplementary Figure 1b is apparently feasible.

The transition to the type-II phase is led independently by the intra-hoping terms of the elements in the secondary chains (i.e. Supplementary Equation 11 depends solely on t_s), which occurs above a critical value for the u^{T} velocity, as shown in Supplementary Figure 1h. Introducing the secondary chains does not impact any of the previous conjectures, as the tilt of the Dirac cones and the properties of the u^{D} matrix are completely disentangled.

Supplementary Note 2: Edge states and Zak phase

The solutions to the eigenvector of the 2×2 Dirac Hamiltonian take the following general form

$$|u\rangle = \begin{bmatrix} a \\ b \end{bmatrix} = \begin{bmatrix} e^{-i\varphi(\mathbf{k})} \\ \pm 1 \end{bmatrix}, \tag{21}$$

where it is easy to prove that $\varphi(\mathbf{k}) = h_{12}$ for the simple Hamiltonian of Supplementary Equation 1.

Assuming a terminated lattice, with N unit cells along the x direction (y is periodic), the eigenvector can be defined as

$$|u\rangle = (a_0, b_0, a_1, b_1, \ldots, a_N, b_N, a_{N+1}, b_{N+1}). \tag{22}$$

We expect solutions (i.e. bulk modes) of the subsequent form

$$|\psi\rangle = e^{-i\beta z} e^{-ik_y y} |u\rangle, \tag{23}$$

$$|u\rangle = \sum_{n=1}^{N} e^{-ik_x n} \begin{bmatrix} e^{-i\varphi(\mathbf{k})} \\ \pm 1 \end{bmatrix}, \tag{24}$$

where the k_x harmonic term is, now, part of the eigenvector. If N valid eigenvectors of this form exist, then the solution set of the $2N \times 2N$ Hamiltonian is complete. If less than N exist, then the unresolved solutions must constitute to other types of states, indicating the existence of edge modes in the system[1].

Let us, now, exclusively consider the A-B termination, i.e. when the A and B elements of Fig. 1a form the top and bottom walls, respectively. Employing, the proper boundary conditions in Supplementary Equation 22, we conclude that $b_0 = 0$ and $a_{N+1} = 0$. Under the first condition, we impose solutions of the form $|u\rangle = |u_{k_x}\rangle - |u_{-k_x}\rangle$, and obtain

$$|u\rangle = \sum_{n=1}^{N} \begin{bmatrix} \sin[k_x n - \varphi(k_x, k_y)] \\ \pm \sin(k_x n) \end{bmatrix}. \tag{25}$$

Utilizing the second condition we finally result in

$$k_x(N+1) - \varphi(k_x, k_y) = \kappa\pi, \qquad \kappa = 1, \ldots, N. \tag{26}$$

This equation suggests that the total number of solutions of the form of Supplementary Equation 23 is equal to the total number of intersections of $\varphi(\mathbf{k})$ with the lines $k_x(N+1) - \kappa\pi$ for $\kappa = 1, \ldots, N$. From the line plots in Supplementary Figure 2, it is deduced that a pair of valid edge states appears for all k_y, where $\varphi(\mathbf{k})$ is discontinuous along the k_x axis. These discontinuities always emerge from the Dirac points and are the source of their non-trivial topological index (if a $\varphi(\mathbf{k})$ integral is calculated on the C curve of Supplementary Figure 2b). In such a case, there are always two lines that do not intersect with $\varphi(\mathbf{k})$. In Supplementary Figures 2a and 2b, the non-intersected blue lines appear in pairs representing the bottom and top edge states.

Supplementary Note 3: Ribbon structures of the chained lattice

The type-I chained lattice comprises a total of 14 elements, 12 of which form the main connective chains. According to the tight-binding set of solution, a pair of bearded edge states will emerge for an A-B terminated lattice. Introducing the 12-chain elements disrupts this assumption, as it cannot be clear anymore whether any of these should exist at the edge. Theoretically, this is resolved by defining a proper termination as the termination that imposes a proper completion of the Dirac spinor eigenmode at the edge. In the binary model, an eigenmode is defined as either a symmetric or an antisymmetric solution, indicated by the sign of Supplementary Equation 20. Expanding the 2×2 Hamiltonian to a multi-element representation (herein, a 14×14 matrix), spreads the two parts of the symmetric/antisymmetric solution, from the two central elements (A

and B), towards all auxiliary nodes of the intermediate chains, constructing a 14-vector solution. This new eigenvector can, then, be separated into two subvectors, u_{A} and u_{B}, by dividing the lattice into two 7-element parts. These subvectors constitute the two parts of a new effective "symmetric/antisymmetric" solution and must remain complete at the edge. Analyzing the bulk system around the Dirac points, hints the actual form of these subvectors and indicates the proper way of termination (Supplementary Figure 3h). In Supplementary Figure 3, we present the band structures of the chained photonic lattice for all possible vertical terminations. In these results we highlight two case that we define as 'proper'. For these cases, a chain is completed with all four elements at the edge.

The first modal group (top subfigure) inherits the topological properties of the bulk band structure though the Zach phase, exhibiting the rich set of type-I and type-II edge states. This modal group is used for the experiments of the main text. In the example of Supplementary Figure 3b (for a $N = 10$ unit cell lattice), we identify the different modes as follows

- Modes 1-8: $$e^{-i\frac{n\pi}{2N}n_{\mathrm{cell,A/B}}}\begin{bmatrix} e^{-i\varphi(k_y)} \\ 1 \end{bmatrix}$$

- Modes 11-18: $$e^{-i\frac{(2N-2-n)\pi}{2N}n_{\mathrm{cell,A/B}}}\begin{bmatrix} e^{-i\varphi(k_y)} \\ -1 \end{bmatrix}$$

where n is the mode index and $n_{\mathrm{cell,A/B}}$ the unit cell and sublattice index (up to $2N$). These are the bulk modes associated with the symmetric and antisymmetric spinor solutions. Moreover,

- Modes 9 and 10: These are the edge state according to the topological definition of the binary lattice and Supplementary Note 2. Note that these are still not specified as proper, a definition reserved for the states of Supplementary Figures 3i and 3j.

- Modes 19-29: An extra set of edge states that complete the set of $2N$ modes of the first modal group. These are outside the theoretical framework of Supplementary Note 2.

Supplementary Note 4: Propagation of light in a waveguide array of couple waveguides

In the photonic lattice of Fig. 1, each individual waveguide constitutes a localized perturbation to the refractive index n_0 of a background dielectric material. In order to describe such a system, we consider the Helmholtz equation from electromagnetics and apply the paraxial approximation by imposing solutions that propagate almost entirely towards the z axis (i.e. the propagation axis of the waveguide array), at a light wavelength of λ_0[2]. Through this process, we extract an equation that describes the envelope ε of the electromagnetic field[3], given by

$$\nabla_{\perp}^2\varepsilon + 2jk_0n_0\frac{\partial\varepsilon}{\partial z} + 2k_0^2n_0\Delta n\varepsilon = 0. \tag{27}$$

By normalizing the spatial coordinates to the dimensions of the unit cell (w_0 for the square lattice) we obtain the following equation

$$\nabla_{\perp}^2\varepsilon + j\left(\frac{2k_0n_0}{z_0}w_0^2\right)\frac{\partial\varepsilon}{\partial z} + (2k_0^2n_0\Delta n w_0^2)\varepsilon = \nabla_{\perp}^2\varepsilon + +j\frac{\partial\varepsilon}{\partial z} + V\varepsilon = 0, \tag{28}$$

$$V = 2k_0^2n_0\Delta n w_0^2, \tag{29}$$

where z_0 indicates the normalization constant of the propagation axis and Δn the refractive index difference between the waveguides and the background material. Note that the second form is obtained by imposing the $z_0 = 2k_0n_0w_0^2$ condition on the second term and is isomorphic to the Schrödinger's equation if we identify z as the dimension of time. This draws a direct analogy between the photonic lattice and a molecular system with atomic sites defined at the waveguide locations where $\Delta n > 0$. A transition to the tight-binding formalism of Supplementary Equations 1-3 is obtained in the weak coupling regime or when V in Supplementary Equation 28 is sufficiently large[2] (within the limits of single mode operation for Δn).

Assuming the validity of all previous assumptions, we expect the emergence of all phenomena associated with the extended relativistic model. In the band diagrams shown in the main manuscript, we directly solve the eigenvalue problem of Supplementary Equation 27, assuming Floquet-Bloch solutions of the form

$$\varepsilon = u(x,y)e^{i(k_xx+k_yy)}e^{ik_zz}, \tag{30}$$

---

[2]Each waveguide is designed for single-mode operation. By means of this condition, we can study the system through a scalar wave equation for $E(x,y,z,t)$.

[3]$E(x,y,z,t) = \varepsilon(x,y,z)e^{j(k_0z-\omega t)}$.

where u follows the periodicity of the square grid. Herein, the eigenvalue k_z assumes the role of energy (E) in the time-independent Schrödinger's equation. As energy levels are closely tied to the potential V, the magnitude of k_z will, also, be related to the wavelength of light k_0, the background index n_0, and the index difference Δn through Supplementary Equation 29. By globally varying any of these parameters (uniformly for all waveguides in the unit cell), the potential V and, consequently, the dispersion diagrams will be scaled by an appropriate constant. This justifies the use of k_0, in Figure 3, as a means to readjust the overall coupling strength between the elements in the lattice. The relativistic dynamics of equation (1) will be revealed by the group velocity $u_{\mathrm{g}} = \partial k_z/\partial \mathbf{k}$, (or $\partial k_z/\partial k_y$ for the terminated lattice). The actual form of u_{g} is tied to the local form of V, which, as a function of the spatial coordinates, is related to the local parameters of each individual waveguide. The tight binding model is able to express this correspondence by associating each hoping term t_i with the index difference Δn of the waveguides in the ith chain.

Supplementary Note 5: Experimental methods

The photonic lattices employed in our experiments were fabricated by focusing ultashort laser pulses from a Ti:sapphire regenerative amplifier system (Coherent Mira/RegA, wavelength 800 nm, repetition rate 100 kHz, pulse length 130 fs) into the volume of a fused silica sample (Corning 7980, dimensions 1 mm × 20 mm × 100 mm, background refractive index $n_0 = 1.457$ at 633 nm), thereby inducing permanent refractive index changes along arbitrary three-dimensional trajectories as defined by the motion of a precision translation system (Aerotech ALS130, inscription speed 100 mm/min). At 633 nm, the elongated, approximately supergaussian index profiles result in slightly elliptical mode fields measure approximately 6 μm × 8 μm. Estimated from numerically inverting the mode profile, the waveguides exhibit a refractive index contrast on the order of $\Delta n_0 = 5 \cdot 10^{-4}$, relative to which the desired detunings were implemented by varying the inscription speed. The sample length is 100 mm and the inscribed lattices are comprised of three by six unit cells, respectively. Subsequently, the Dirac dynamics of the system were studied by probing specific sites with single-waveguide excitations, and observing the resulting discrete diffraction pattern at the end of the sample. Using a whitelight source (NKT SuperK Extreme), we were able to seamlessly tune the excitation wavelength while maintaining a bandwidth of approximately 2 nm.

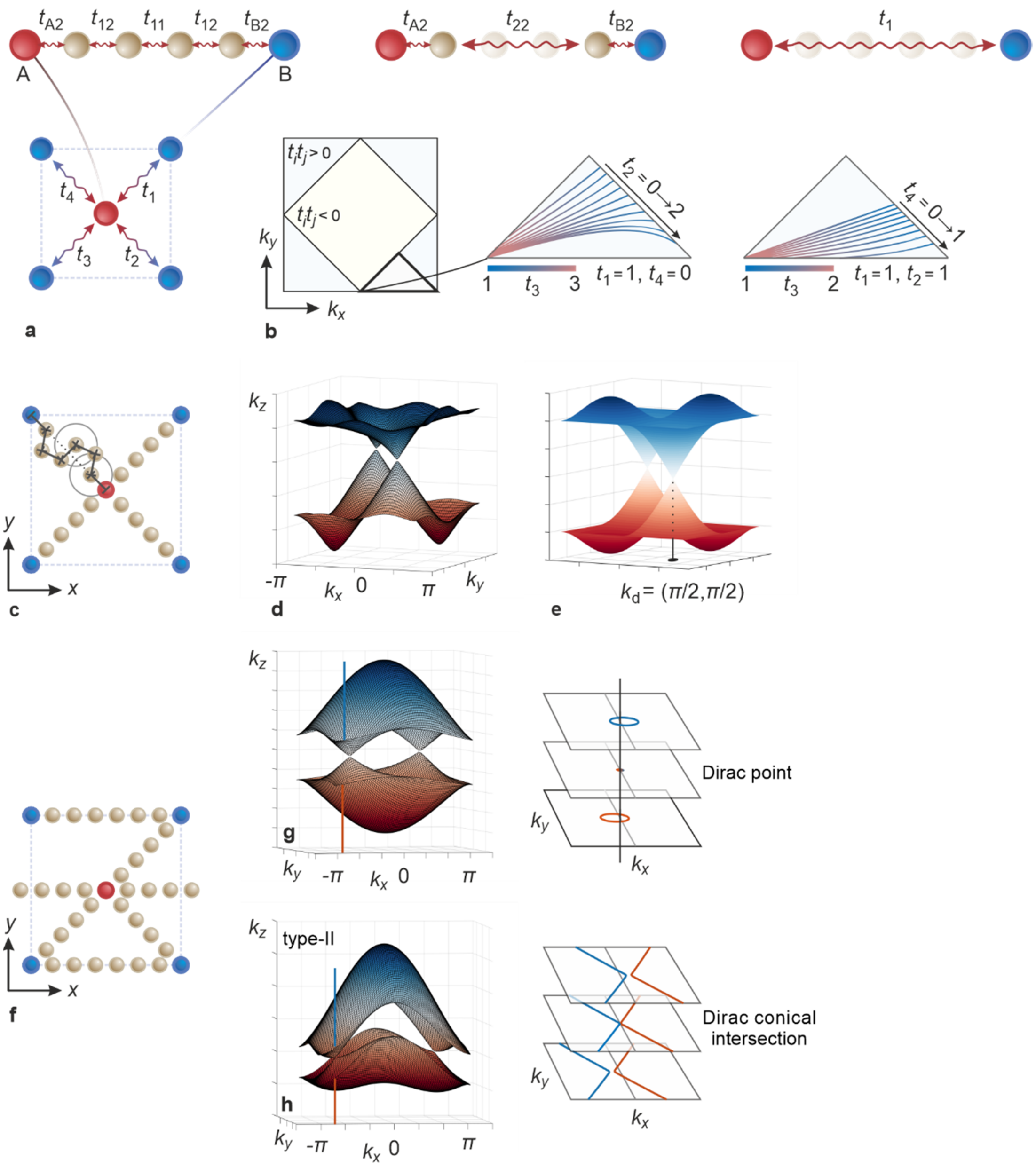

Supplementary Figure 1 | The ideal 2 × 2 Hamiltonian and its chained lattice implementations. a, A regular 4-element chain is defined by 4 independent hoping terms. Applying Supplementary Equations 4 and 5 twice, we extract the effective term t_1, at β_D (k_{zD}). **b,** The solution set of Supplementary Equations 9 divides the Brillouin zone into two sections. The inner section can only be accessed when a hoping term displays a reversed sign. Two groups of parametric perturbations are shown on the right. The rest of the outer **k**-space is covered by all transformations defined by the spatial symmetries of the square lattice. **c,** A 4-chain lattice with 6 elements in the fourth main chain. **d,** Band diagram of **c** depicting two inner bands. **e,** Band diagram of the 2 × 2 Hamilotian with $t_1 = t_2 = t_3, t_4 = -t_1$. **f,** The chained type-II square lattice. Band diagrams and intersections with constant k_z planes for **g,** below and **h,** just above the type-II phase transition threshold.

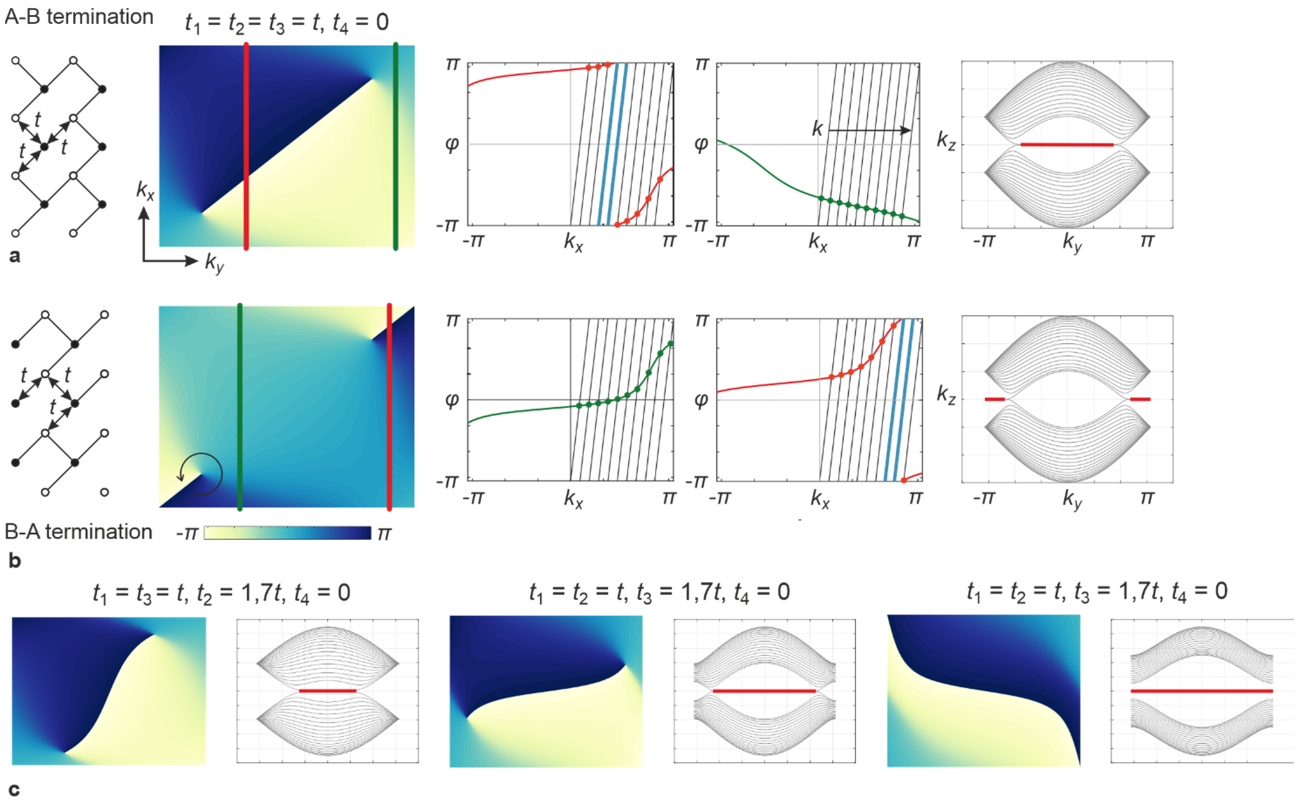

Supplementary Figure 2 | Zach phase and edge states in the square anisotropic lattice. a, The A-B termination of the 3-chain isotropic lattice generates a pair of edge states at $|k_y| < 2\pi/3$. The $\varphi(\mathbf{k})$ curves, for two constant k_y lines, are drawn in the middle. The two blue lines correspond to unresolved solutions of Supplementary Equations 26, indicating the presence of the two degenerate edge states. **b,** A similar characterization for the B-A termination where the pair of edge states emerges at $|k_y| > 2\pi/3$. **c,** Three different perturbations resulting in the relocation of the Dirac degeneracies and the variation of the topological phase.

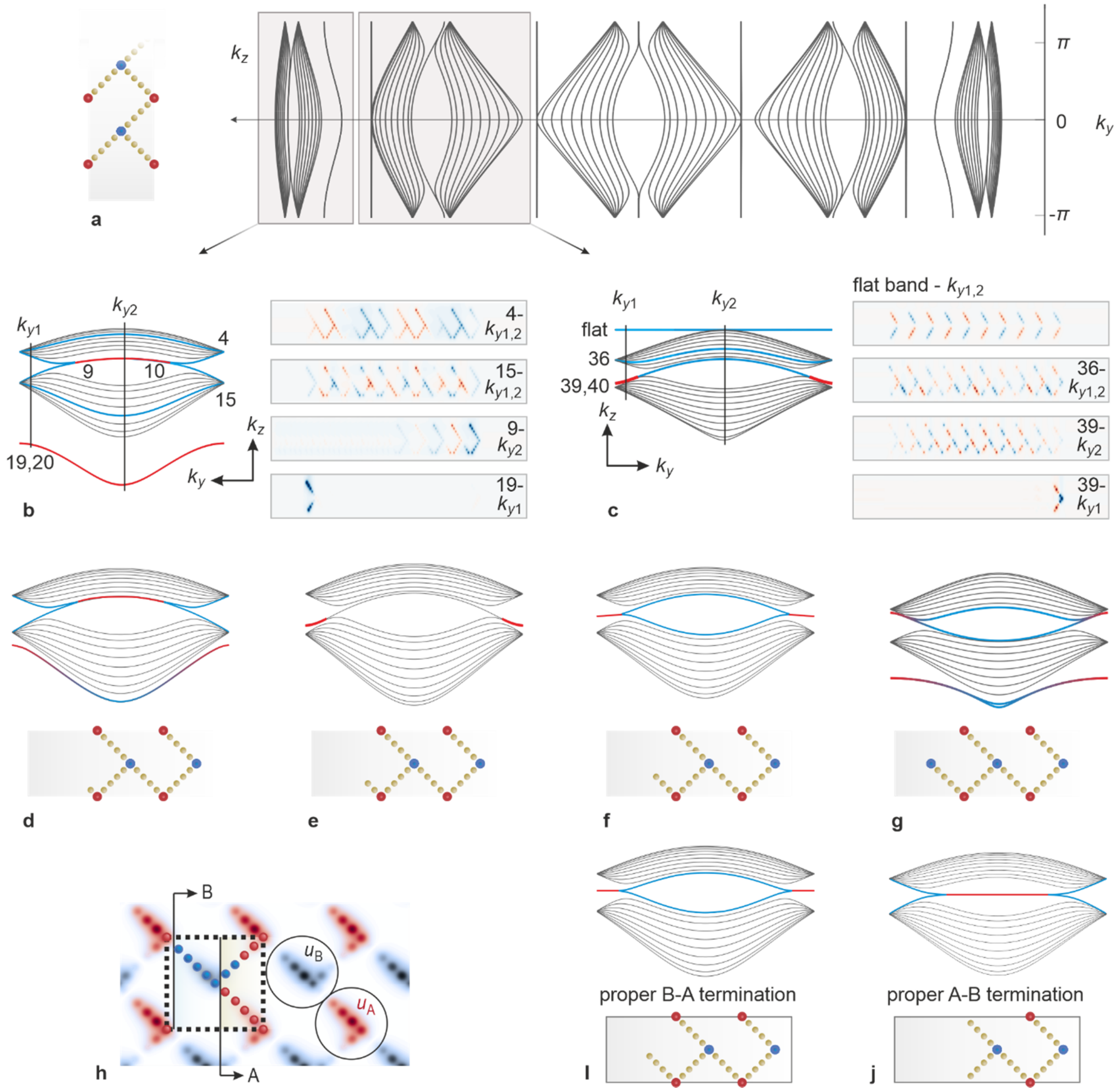

Supplementary Figure 3 | Edge states of the terminated chained lattice. a, An attempt to terminate the lattice in its central nodes and the respective full band diagram. **b,** The first group of modes does not produce the expected pair of bearded edge states. **c,** the secondary group produces states that do not conform to the proper topological characterization and are distinct from **b**. **d-g,** A number of cases towards the search for the proper termination. **h,** the bulk eigenvector can be separated into two subvectors u_A and u_B, near the Dirac points in the **k**-space. **i,j,** the proper A-B and B-A terminations result in the topologically characterized states. The secondary groups of modes, not depicted here, follow an identical characterization to the first.

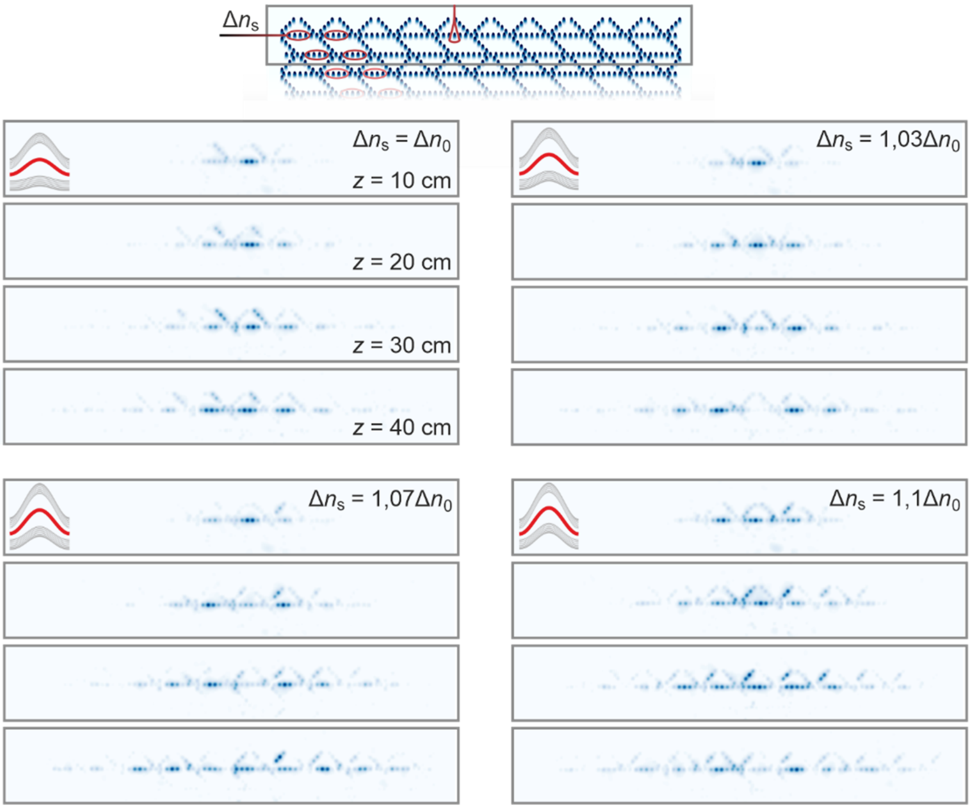

Supplementary Figure 4 | Simulation results of the type-II lattice for various detuning magnitudes of the secondary chains. A direct comparison can be made with the experimental results of Fig. 3.

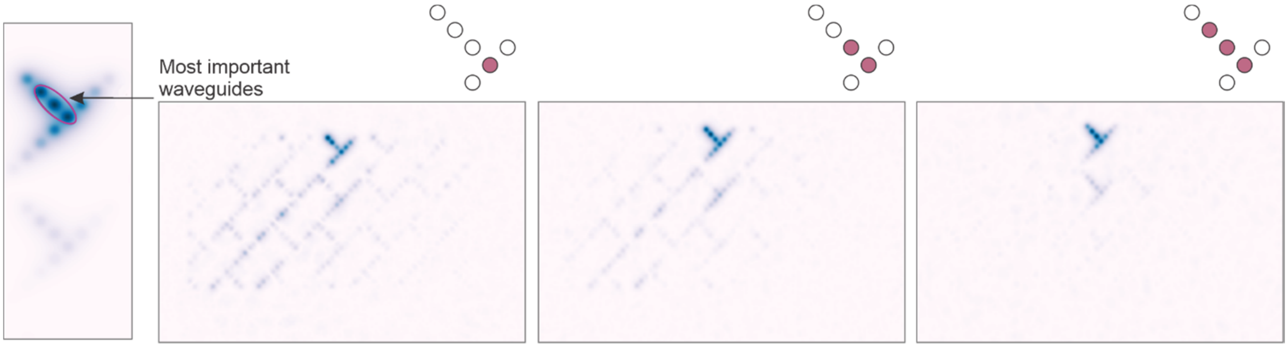

Supplementary Figure 5 | Simulation study of different excitations. The edge eigenmode is shown on the left. We indicate the "most important" waveguides as the three sites that confine the majority of light power. Results demonstrate the output facet at a distance *z* similar to the experimental, for a single, double, and triple waveguide excitation, as we progressively attain better matching and less leakage. In our experiments, we experience leakage levels similar to the second case by exciting the middle waveguide.

Supplementary References

1. Delplace, P., Ullmo, D. & Montambaux, G. Zak phase and the existence of edge states in graphene. *Phys. Rev. B* **84**, 195452 (2011).
2. Yariv, A. & Yeh, P. *Photonics: Optical Electronics in Modern Communications*. (Oxford University Press, 2007).
