# Supplementary material for: Symmetry-controlled edge states in the type-II phase of Dirac photonic lattices
Source: Nat Commun. 2020 Apr 29;11:2074. doi: 10.1038/s41467-020-15952-z (PMC7190735; doi:10.1038/s41467-020-15952-z)
Supplement: Supplementary file 3 — Description of Additional Supplementary Files [file 41467_2020_15952_MOESM3_ESM.pdf]

**Description of Additional Supplementary Files**

**File Name:** Supplementary Data 1

**Description:** A data sheets file with the experimental data of Figure 3. Individual tabs contain the lattice site coordinates and the surface plot data that reproduce the respective subfigures.
